# Genotyping by RAD Sequencing Analysis Assessed the Genetic Distinctiveness of Experimental Lines and Narrowed down the Genomic Region Responsible for Leaf Shape in Endive (*Cichorium endivia* L.)

**DOI:** 10.3390/genes11040462

**Published:** 2020-04-23

**Authors:** Alice Patella, Fabio Palumbo, Samathmika Ravi, Piergiorgio Stevanato, Gianni Barcaccia

**Affiliations:** Department of Agronomy, Food, Natural Resources, Animals and Environment (DAFNAE), University of Padova, 35020 Legnaro PD, Italy; alice.patella@phd.unipd.it (A.P.); fabio.palumbo@unipd.it (F.P.); samathmikaravi@gmail.com (S.R.); stevanato@unipd.it (P.S.)

**Keywords:** RAD-seq, SSR markers, SNP markers, genetic diversity, *ELO2/AtELP1*, leaf shape, endive, breeding

## Abstract

The characterization of genetic diversity in elite breeding stocks is crucial for the registration and protection of new varieties. Moreover, experimental population structure analysis and information about the genetic distinctiveness of commercial materials are essential for crop breeding programs. The purpose of our research was to assess the genetic relationships of 32 endive (*Cichorium endivia* L.) breeding lines, 18 from var. *latifolium* (escarole) and 14 from var. *crispum* (curly), using heterologous *Cichorium intybus*-derived simple sequence repeats (SSR) markers and single-nucleotide polymorphisms (SNP) markers. We found that 14 out of 29 SSR markers were successfully amplified, but only 8 of them were related to polymorphic loci. To overcome the limitation of the low number of informative SSR marker loci, an alternative SNP-based approach was employed. The 4621 SNPs produced by a restriction site-associated DNA marker sequencing approach were able to fully discriminate the 32 endive accessions; most importantly, as many as 50 marker loci were found to distinguish the curly group from the escarole group. Interestingly, 24 of the marker loci mapped within a peripheral segment of chromosome 8 of lettuce (*Lactuca sativa* L.), spanning a chromosomal region of 49.6 Mb. Following Sanger sequencing-based validation, three genes were determined to carry nonsynonymous SNPs, and one of them matched a putative ortholog of *AtELP1*, subunit 1 of the Elongator complex. Considering that several previously characterized Elongator complex subunit mutants exhibited elongated and/or curly leaf phenotypes, this gene should be taken into consideration for a better understanding of the underlying mechanism controlling leaf shape in endive.

## 1. Introduction

Endive (*Cichorium endivia* L., 2*n* = 2*x* = 18) is a leafy green vegetable belonging to the Asteraceae family [1,2]. It can be further classified into two cultivar types: curly endive (*C. endivia* var. *crispum* Lam.) and escarole, or smooth, endive (*C. endivia* var. *latifolium* Lam.) [3]. From a reproductive point of view, endive is an autogamous species with a rate of outcrossing of approximately 1%; therefore, natural populations are composed of a mixture of highly homozygous lines [4,5]. This species, along with lettuce and chicory, is widely utilized for the preparation of ready-to-use salads, which are being consumed in increasing amounts due to their healthy properties. Several biological activities and properties are attributed to this crop, such as anti-inflammatory, antioxidant, and hepatoprotective properties [6,7]. Consequently, a rising interest in ready-to-eat food with health benefits is leading seed companies to invest in breeding programs and variety protection in endive. Typically, commercialized endive cultivars mostly consist of pure lines [8], and the risk of elite genotype plagiarism is very high. To make matters worse, the International Seed Federation (ISF) requires the holder of the initial variety (and not the alleged plagiarist) to provide the proof to resolve any legal dispute. Therefore, the genetic characterization of any new registered variety plays a key role in its legal protection [9]. Depending on the species, different coefficients are used and similarity thresholds are set to genetically differentiate the varieties (i.e., in lettuce, the coefficient is Jaccard, and the threshold is 96%) [10]. Moreover, the genetic characterization of a new variety also enables the verification of its distinctiveness, uniformity, and stability (DUS testing), which are three major requirements for its registration. Currently, molecular markers, among their other advantages, represent useful tools for both the registration procedure and the legal protection of cultivars. Due to their codominant nature, high frequency in all genomes, and high reproducibility among laboratories, the most commonly used markers are simple sequence repeats (SSRs) [11] and single-nucleotide polymorphisms (SNPs) [9]. However, to date, the only source of molecular data available for *C. endivia* derives from a transcriptomic analysis performed by Testone et al. [5]. The most studied species in the genus *Cichorium* is instead *C. intybus* (leaf chicory) due to the availability of a draft genome [12] and some relevant molecular assays pertaining to breeding [13,14,15,16]. In particular, for *C. intybus,* an informative panel of SSR markers [14,15] was demonstrated to be very effective for the genetic characterization of both hybrid parental materials and synthetic varieties. Another research study provided a genetic consensus map for *Cichorium spp*. [17] that includes markers from a *C. intybus* × *C. endivia* cross [18].

Starting with the molecular information available for leaf chicory and the close genetic relationship between this species and *C. endivia,* we first tried to characterize a pool of 32 elite endive lines using heterologous chicory-derived microsatellites, evaluating their intra-genus transferability. The same samples were also analyzed by means of a restriction site-associated DNA marker (RAD) sequencing approach, and the resulting SNP markers were used to discriminate the 32 endive lines and to predict the two main cultivar types (var. *latifolium* and var. *crispum*). Finally, taking advantage of the newly developed SNP set, we successfully narrowed down the genomic region responsible for the curly trait.

## 2. Materials and Methods

### 2.1. Plant Materials

Thirty-two experimental lines of endive (F5) belonging to Blumen Group SpA, Italy, were used in this study. Specifically, 18 samples (numbered from 1 to 18) belonging to *C. endivia* var. *latifolium* (escarole or smooth endive) and 14 individuals (numbered from 19 to 32) of *C. endivia* var. *crispum* (curly endive) were analyzed. Genomic DNA was isolated from 100 mg of fresh leaves using a DNAeasy plant mini kit (Qiagen, Valencia, CA, USA) following the procedure provided by the manufacturer. Both the quality and quantity of the genomic DNA samples were assessed by agarose gel electrophoresis (1% agarose/1× TAE gel containing 1× Sybr Safe DNA stain, (Life Technologies, Carlsbad, CA, USA) and a NanoDrop 2000c UV-Vis spectrophotometer (Thermo Fisher Scientific Inc., Pittsburgh, PA, USA), respectively.

### 2.2. SSR-Based Genotyping by Heterologous Chicory-Derived Microsatellites

Three random samples diluted to 15 ng/µL were first amplified using 29 heterologous SSR primer couples of *C. intybus* [15] to evaluate their transferability within the genus. The microsatellite loci were first tested individually, and those producing specific amplicons were further organized in three multiplex reactions (Table 1). PCRs were performed following the method described by Schuelke et al. [19], with some variations. Briefly, three primers were used to amplify each microsatellite locus: a pair of locus-specific primers, one of which had an oligonucleotide tail at the 5’ end (PAN-1, PAN-2, PAN-3 and M13, Table S1), and a third primer (from now on called the “universal primer”) complementary to the tail and labeled with a fluorescent dye (VIC, NED, PET and 6-FAM, respectively).

All PCRs were performed in a 10 µL reaction volume containing 1× Platinum^®^ Multiplex PCR Master Mix (Thermo Fisher Scientific Inc., Waltham, MA, USA), 10% GC enhancer (Thermo Fisher Scientific Inc., Waltham, MA, USA), 0.25 µM of the non-tailed primer, 0.75 µM of the tailed primer, 0.5 µM of the universal primer, and 15 ng of genomic DNA. The thermal cycling conditions were as follows for all multiplexes: 94 °C for 5 min, followed by 8 cycles of 94 °C for 30 s, 61 °C for 30 s, 72 °C for 30 s; with an annealing temperature stepdown every cycle of 1 °C (from 61 to 54 °C). The annealing temperature for the following 37 cycles was set to 54 °C, with denaturation and extension phases as above and a final extension at 60 °C for 30 min. The PCR products were amplified in a GeneAmp^®^ PCR 9700 thermal cycler (Applied Biosystems, Carlsbad, CA, USA). The amplifications were first tested with gel electrophoresis (2% Ultrapure™ Agarose in TAE 1×, SYBR Safe^®^ 1×, (Life Technologies, Carlsbad, CA, USA) and then run with capillary electrophoresis with an ABI 3730 DNA analyzer (Applied Biosystem, Carlsbad, CA, USA), adopting LIZ500 as the molecular weight standard. The size of each peak was determined using the Peak Scanner software 1.0 (Applied Biosystems, Carlsbad, CA, USA).

For those markers that produced amplicons, the polymorphic information content (PIC) was calculated using the POPGENE software [20]. The UPGMA dendrogram and principal coordinate analysis (PCoA) centroids were constructed by applying Jaccard’s coefficient and plotted using the PAST software v. 3.14 with 1,000 bootstrap repetitions [21]. Finally, a Bayesian clustering algorithm implemented in STRUCTURE v.2.2 [22] was used to model the genetic structure of the endive core collection. The number of founding groups ranged from 1 to 15, and 10 replicate simulations were conducted for each value of K, setting a burn-in of 200,000 and a final run of 1,000,000 Markov chain Monte Carlo (MCMC) steps. STRUCTURE HARVESTER [23] was used to estimate the most likely value of K, and the estimates of membership were plotted as a histogram using an Excel spreadsheet.

### 2.3. SNP-Based Genotyping by RAD-Seq Analysis

The same 32 endive samples used for the SSR analysis were also analyzed by means of a restriction site-associated DNA marker (RAD) sequencing approach. One microgram of DNA per individual was digested with the restriction enzymes *Pst*I and *Msp*I (New England Biolabs, Ipswich, MA, USA), following the procedure by Stevanato et al. [24]. For the library preparation, digested DNAs were diluted at concentrations of 3.0 ng/µL. The library preparation, sequencing run, and bioinformatics analyses were carried out according to the protocol described by Stevanato et al. [24]. Raw reads obtained through an Ion S5 sequencer (Thermo Fisher Scientific Inc., Waltham, MA, USA) were trimmed according to the enzyme recognition sequence, and after a quality check, all the artifacts and the Ns-containing reads were removed. Variants were called using the Stacks v2.41 software [25], and SNPs were filtered to remove those meeting the following criteria: (1) SNPs with more than 10% missing data, (2) SNPs with a sequence depth ≤ 4, and (3) tri- and tetra-allelic SNPs.

The UPGMA dendrogram, principal coordinate analysis (PCoA), and genetic structure study of the endive core collection were performed as already described for the SSR markers, while the observed homozygosity values were estimated with the POPGENE software [20]. SNP-containing reads were annotated against the genome of lettuce (*Lactuca sativa*), retrieved from Phytozome [26], and derived from Chin-Wo S et al. [27]. A local BLASTn-based approach (E-value ≤ 1.0 × 10^−7^, BLAST+ v.2.3.0) was used.

### 2.4. SNP Marker Validation

The six reads able to discriminate the curly samples (var. *crispum*) from the escarole samples (var. *latifolium*) and carrying nonsynonymous mutations were further investigated in a Sanger sequencing step. This was achieved by anchoring and annotating the endive reads on the draft genome of leaf chicory [12] using a local BLASTN approach (E-value ≤ 1.0 × 10^−7^, BLAST+ v.2.3.0). Primer pairs were then designed on chicory contigs to produce predicted amplicons between 200 bp (i.e., read 414) and 700 bp (i.e., 3614) to span the read-containing genic regions (Table S2). PCRs were performed on 16 samples (8 from var. *crispum* and 8 from var. *latifolium*) in a GeneAmp^®^ PCR 9700 thermal cycler (Applied Biosystems, Carlsbad, CA, USA) with the following conditions: initial denaturation at 95 °C for 5 min, followed by 40 cycles at 95 °C for 30 s, 56 °C for 30 s, 72 °C for 40 s, and a final extension of 10 min at 72 °C. The quality of the PCR amplicons was assessed on a 1.5% (w/v) agarose gel stained with 1× SYBR Safe ^TM^ DNA Gel Stain (Life Technologies, Carlsbad, CA, USA). Amplicons were purified with ExoSap-IT (Applied Biosystems, Carlsbad, CA, USA), sequenced through Sanger sequencing, analyzed in Geneious 11.1.4 (http://www.geneious.com, [28]), and deposited in GenBank (accession No. from MT166347 to MT166352 and from MT177272 to MT177283). 

## 3. Results

### 3.1. SSR-Based Genotyping by Heterologous Chicory-Derived Microsatellites

In a preliminary analysis aimed at investigating the transferability of SSR markers among species of the *Cichorium* genus, 14 out of 29 heterologous microsatellite primer pairs (48%) retrieved from *C. intybus* [15] were found to generate amplicons. The 14 SSR markers were then organized in three multi-locus PCRs and used to genotype the whole set of samples (32 individuals). The number of polymorphic microsatellites was eight, according to Botstein et al. [29], of which four had PIC values that were highly informative (≥0.50) (from 0.58 to 0.70), and the other 4 were reasonably informative (0.42 < PIC < 0.50) (Table S3). 

**Figure 1:**
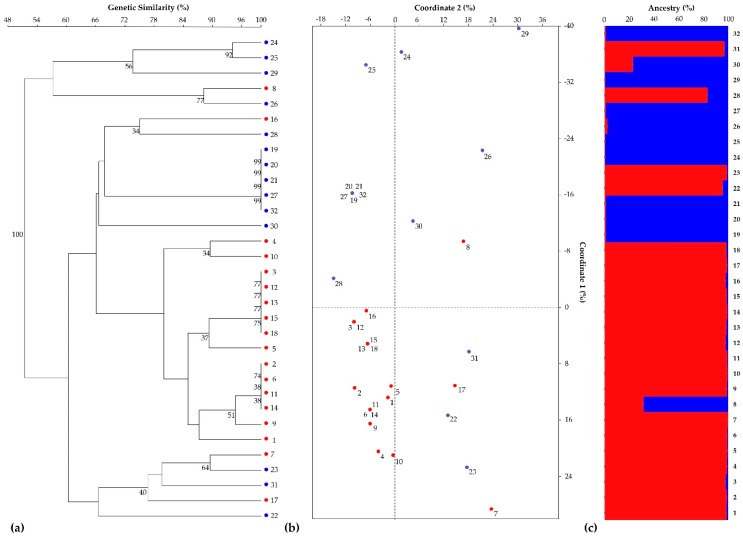
Grouping analysis of 32 accessions of endive based on 14 SSR markers. (**a**) UPGMA dendrogram of genetic similarity estimates computed among pairwise comparisons of individual samples using Jaccard’s coefficient. Bootstrap estimates ≥ 30% are reported next to the nodes (red and blue dots highlight the two endive cultivar types, *C. endivia* var. *latifolium* and *C. endivia* var. *crispum*). (**b**) Principal coordinate analysis (PCoA) centroids derived from the analysis of genetic similarity estimated with Jaccard’s coefficient (red and blue dots correspond to the individual samples of the two cultivar types, *C. endivia* var. *latifolium* and *C. endivia* var. *crispum*). (**c**) Population genetic structure of the 32 endive samples as estimated by STRUCTURE. Each sample is represented by a vertical histogram partitioned into *K* = 2 colored segments (red or blue, corresponding to (**a**) and (**b**)) representing the estimated membership. The proportion of ancestry (%) is reported on the ordinate axis, and the identification number of each accession is reported below each histogram.

The UPGMA dendrogram constructed with Jaccard’s coefficient divided the samples into three main clusters, with bootstrap supports always lower than 50%, except for a few nodes (Figure 1a). 

From the PCoA, the first principal coordinate accounted for 35% of the total variation and separated the samples into two groups, while the second coordinate accounted for 16% of the total variation (Figure 1b). The UPGMA dendrogram and PCoA centroids did not fully distinguish the endive accessions or the two endive cultivars (Figure 1b). From the genetic structure analysis, following the procedure of Evanno et al. [22], a clear maximum for the Δ*K* value at *K* = 2 was found (Δ*K* = 240, Figure S1). Although the population size *K* = 2 also corresponds to the number of varieties used in this study (var. *crispum* and var. *latifolium*), the estimated membership of each sample to the ancestral genotypes did not reflect the subspecies classification, as already shown by the UPGMA dendrogram and the PCoA centroids (Figure 1c).

### 3.2. SNP-Based Genotyping by RAD-Seq Analysis

A RAD-seq analysis was performed using the same 32 samples already tested using 14 heterologous SSR primer couples. The Ion S5 sequencing produced 81,200,000 raw reads, on average 2,030,891 ± 150 per sample. After quality and adapter trimming, we obtained 72,670,760 reads that were used to create a catalog of 18,806 consensus loci, used as reference for variant calling. A raw pool of 6242 SNPs was first identified; after the filtering step, 4621 SNPs distributed in 4482 RAD sequence tags were retained. 

**Figure 2:**
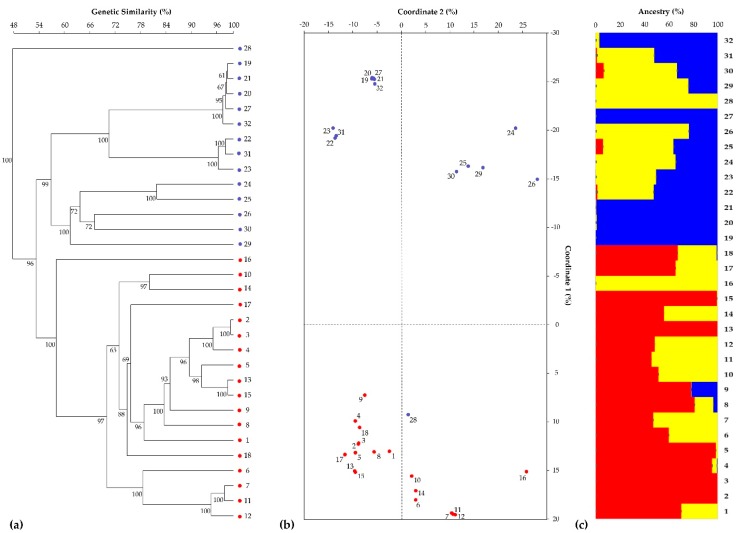
Grouping analysis of 32 endive samples based on 4621 SNP markers. (**a**) UPGMA dendrogram of genetic similarity estimates computed among pairwise comparisons of individual samples using the Jaccard coefficient. Bootstrap estimates ≥ 30% are reported next to the nodes (red and blue dots highlight the two endive cultivar types, *C. endivia* var*. latifolium* and *C. endivia* var*. crispum*). (**b**) PCoA centroids derived from the analysis of genetic similarity estimated with Jaccard’s coefficient (red and blue dots correspond to the individual samples of the two cultivar types, *C. endivia* var*. latifolium* and *C. endivia* var*. crispum*). (**c**) Population genetic structure of the 32 endive samples as estimated by STRUCTURE. Each sample is represented by a vertical histogram partitioned into *K* = 3 colored segments (red, blue, corresponding to (**a**) and (**b**), or yellow) representing the estimated membership. The proportion of ancestry (%) is reported on the ordinate axis, and the identification number of each accession is indicated below each histogram.

The average similarity values calculated in all possible pairwise comparisons between all samples are reported in Figure S2 and overall ranged from 52.0% (sample 28 vs. sample 23) to 99.7% (sample 2 vs. sample 3, distinguishable for just 23 SNPs). When calculated within two cultivar types, these values varied from 63.9% to 99.7% (mean = 83.9%) in the escarole endive and from 52.0% to 99.5% (mean = 74.9%) in the curly endive. In the pairwise comparisons between the escarole and curly type accessions, the average similarity values (67.7%) ranged from a minimum of 57.1% to a maximum of 78.4%. The first two components of the PCoA were able to together explain 53.8% of the molecular variation, accounting for 36.7% and 17.0%, respectively (Figure 2b), revealing a tight aggregation among samples of the same cultivar type and, at the same time, a clear-cut separation between the curly and escarole types. This latter aspect is also particularly evident in the UPGMA tree (Figure 2a). It is also worth mentioning that, both in the UPGMA dendrogram and the PCoA centroids, sample 28 and, to a lesser extent, sample 16 clustered completely apart from the rest of the samples.

From the STRUCTURE analysis, the highest Δ*K* value was found at *K* = 3 (Figure S3), suggesting three different common ancestors. More specifically, one ancestor (Figure 2c, see blue segments) was shared by the curly endive samples, while a second putative ancestor (Figure 2c, see red segments) characterized all the escarole individuals. Furthermore, both cultivar types had cases of admixed individuals, with a variable percentage of membership in a third cluster (Figure 2c, see yellow segments). It is noteworthy that none of the admixed individuals showed simultaneous membership in both main ancestor clusters (except, partially, sample 9). Finally, samples 16 and 28 were the only ones derived completely (100%) from the third ancestor (Figure 2c).

The observed homozygosity computed across all individual DNA accessions ranged from 74.2% to 97.6%, with an average estimate of 95.9 ± 4.1% (Figure S2).

**Figure 3:**
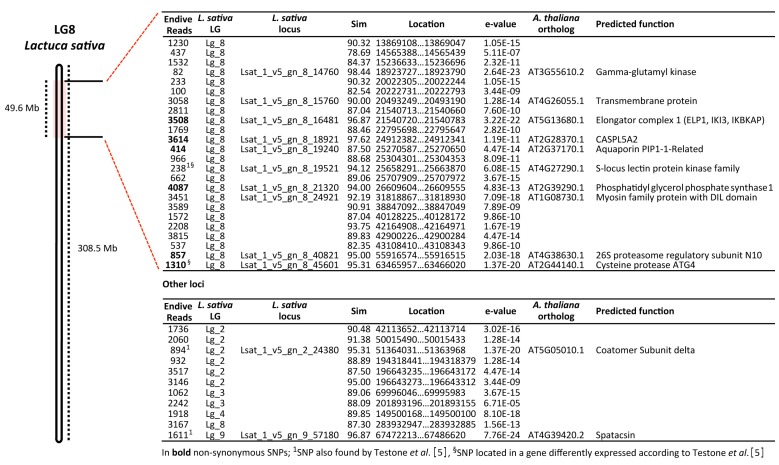
Information on the reads that discriminate escarole from curly endive. The name of the endive single-nucleotide polymorphisms (SNP)-containing reads, *Lactuca sativa* linkage group (LG), genic locus, similarity location and E-value are reported along with the best *Arabidopsis thaliana* match and the predicted function. Twenty-four endive reads were mapped within a peripheral segment of chromosome 8 of *L. sativa,* spanning a DNA region of 49.6 Mb.

From the BLASTn analysis (E-value ≤ 1.0 × 10^−7^) performed by aligning the 4482 SNP-carrying sequences against the lettuce exome [27], 555 (12.4%) showed at least one significant match and were uniformly distributed throughout the nine linkage groups of the *L. sativa* genome (Table S4). The average similarity was 95.5%, and 61.8% of matches exhibited similarity scores higher than 95% (Figure S2). Among the 4621 SNPs, 50 markers fully discriminated curly endive from escarole, and 24 of them mapped within a peripheral segment of chromosome 8 of *L. sativa*, spanning a DNA region of 49.6 Mb (Figure 3). From the BLASTn alignment, 10 out of 24 also fell in genic regions. Six of them carried nonsynonymous mutations (3508, 3614, 414, 4087, 857, and 1310, Figure 3), while two were already reported by Testone et al. [5]. Four out of the six loci carrying nonsynonymous SNPs and identified through the RAD-seq analysis were also confirmed in the Sanger sequencing-based validation step. Sanger sequencing also allowed us to identify 27 other single nucleotide variations (SNVs) and 2 INDELs fully discriminating the two endive cultivar biotypes (escarole and curly) (Table S5). Sixteen of them were found in exon regions, and five were nonsynonymous. Among these latter five, three nonsynonymous mutations fell in the putative ortholog of a CASP-like protein (AT2G28370.1), while the other two were detected within the putative orthologs of ELP1 (AT5G13680.1) and phosphatidyl glycerol phosphate synthase 1 (AT2G39290.1).

## 4. Discussion

The deposition of variety genotypes plays a crucial role in the registration and legal protection process of those crop species, such as endive, that are usually characterized by pure lines [8]. However, in *C. endivia*, the total lack of an informative and robust panel of markers makes any genotyping analysis complicated. For this reason, the first aim of this work was to develop a molecular assay for this species able to characterize and fully discriminate a pool of 32 endive (*C. endivia*) lines of a high breeding value (F5 generations).

In the first section, an approach based on the exploitation of heterologous SSR primers retrieved from *C. intybus* [15] was evaluated. Despite the interfertility between *C. endivia* and *C. intybus* and the availability of molecular linkage maps referring to such hybrids [17], only 48% of microsatellites were successfully transferred. Generally, the use of transferable cross species/genera microsatellite markers is considered a cost-effective approach to ensure the ubiquitous applicability of markers in genomic resources [30,31]. However, it has been demonstrated that increasing the genetic distance among taxa may reduce the marker transfer efficiency [32,33]. For example, some recent studies aimed to test the SSR transferability among genera of the same family (e.g., Cactaceae, Arecaceae and Apiaceae) and achieved success rates of approximately 23–35% [34,35,36]. At the genus level, the percentage of SSR transferability among species rises to 80–85% [37,38] or even higher (90–98%) when EST-SSR are considered [39,40]. Thus, the reason for the low intra-genus SSR transferability rate found in this study remains unclear, especially considering the relatively short evolutionary distance between the source (*C. intybus*) and the target species (*C. endivia*) [41] and their interfertility.

The small number of effective microsatellites in combination with the low number of polymorphic microsatellites was not sufficient to discriminate between the two cultivar types or between plant materials of the same cultivar. This lack of informativeness was highlighted by the UPGMA dendrogram and the PCoA centroids. In fact, samples were clustered in three main subgroups that deviated from the expected cultivar types (Figure 1a,b) and, in general, from the morphological observations. Additionally, STRUCTURE analysis showed the limits of the SSR panel because two different ancestors were recognized without completely discriminating escarole from curly endive (Figure 1c).

To overcome some of these limits, a second SNP-based approach was attempted. The RAD-based sequencing used is a well-established and powerful method for recovering thousands of polymorphic loci across the genomes of many crop species [42,43,44]. We identified 4621 biallelic SNP loci (9242 possible alleles) discriminating the escarole and curly varieties of *C. endivia* and substantially increasing the existing knowledge of the genetic diversity of endive. According to the scientific literature, this method usually yields, on average, from 12,000 to 40,000 SNPs depending on the species, the data processing filters, and the inherent genetic diversity in the plant material [24,44,45,46]. The moderate number of SNPs detected in this study is directly related to the specific sequencing technology throughput (Ion Torrent S5), which is significantly lower than that of other sequencing technologies (e.g., Illumina platforms). However, our finding could also be dependent on the small size of the endive genome and on the low levels of genetic diversity in the plant material.

The discriminant SNP markers identified in our work resulted in a valuable tool that allowed us to efficiently discriminate 32 endive samples. RAD-seq data were in fact used to conduct a genetic diversity analysis that showed high levels of genetic similarity overall, as observed at the phenotypic level, especially within escarole (var. *latifolium*) materials (83.9%). Contrary to what emerged from the SSR-based analysis, all samples were also univocally discriminated, and the most similar samples (samples 2 and 3) were distinguishable based on 23 SNPs. In this case, to prevent the registration and the release on the market of two almost identical (and thus essentially derived) varieties, a choice will be made by breeders according to the phenotypic data and to precommercial trials.

Interestingly, the SNP-based STRUCTURE analysis showed three different ancestral groups for the endive materials, highlighting the importance of analyzing plant material that is the result of crosses between different germplasm sources. According to the phenotype information, in the genetic structure analysis, samples were attributed to escarole or curly endive ancestors, except for two samples, namely, 16 and 28, which resulted from a third ancestor. These findings confirmed the UPGMA dendrogram results, in which the two samples appeared as out-groups.

The overall observed homozygosity was, on average, 95.9 ± 4.1%, consistent with the autogamous reproductive system of this species and with the five self-pollinated cycles that these materials had undergone. Since low heterozygosity values guarantee progenies with the desired genetic stability and, consequently, phenotypic uniformity [8], individuals with high homozygosity could be selected to produce precommercial varieties characterized by genetic stability and thus uniformity. The RAD-seq technique also represents an interesting tool for improving the discovery of molecular markers linked to specific traits of interest [47,48,49]. For this reason, the 4621 SNP markers were annotated through a BLASTn approach against the coding regions of *Lactuca sativa*, a key species in the Asteraceae family. Of the SNPs, 12.4% showed at least one significant match. Although coding regions usually represent 2–3% of the entire genome [50,51], this finding is not surprising. In fact, RAD-seq is an extremely versatile method, and the selection of the enzymes used for the preparation of the sequencing libraries allows us to choose whether to focus the analysis more on coding regions or on intergenic regions. In this specific case, the enzyme combination exploited in this work (*Pst*I/*Msp*I) was confirmed to be one of the most effective in producing fragments within genic regions [52]. Another interesting achievement is the successful distribution of the RAD-seq markers throughout the nine linkage groups in lettuce. In fact, regardless of the marker type, good genome representativeness is always strongly suggested to avoid biased estimates of nucleotide diversity and to prevent incorrect estimates of genetic subdivisions [53,54].

One of the major results of this work is that among the 50 SNP markers that were able to fully discriminate curly and escarole endive, 24 of them mapped within a peripheral segment of chromosome 8 of *L. sativa*, spanning a DNA region of 49.6 Mb. Moreover, six of them carried nonsynonymous SNVs (3508, 3614, 414, 4087, 857, and 1310, Figure 3). This finding is extremely meaningful if we consider that nearly half (24/50) of the discriminating SNPs fall in a genomic window (49.6 Mb) that represents less than 2.1% of the entire lettuce genome (2.22 Gb, [55]).

The additional Sanger-based validation step performed on the six loci carrying nonsynonymous mutations confirmed the extraordinary concentration of leaf morphology-related SNPs in this region. In fact, four out of six SNPs were confirmed, and 27 new SNPs and 2 INDELs fully discriminating the two endive cultivar biotypes (escarole and curly) were identified. Most importantly, more than half of the SNPs (i.e., 16) were in exon regions, and five were nonsynonymous variants. The putative orthologs of Lsat_1_v5_gn_8_18921 (*L. sativa*) and AT2G28370.1 (*A. thaliana*), annotated as CASPARIAN STRIP MEMBRANE DOMAIN PROTEIN (CASP)-Like 5A2, showed the highest number of mutations (11 SNPs and 2 INDEL). On the contrary, the putative orthologs of Lsat_1_v5_gn_8_16481 (*L. sativa*) and AT5G13680.1 (*AtELP1, A. thaliana*), and that codifying for the Elongator complex protein 1 (*ELP1/ELO2*), scored the highest number of nonsynonymous SNPs (i.e., 3 SNPs).

Although it has been demonstrated that CASP-like proteins may play an indirect role in leaf growth and development [56,57], the significant lack of information on this family makes it difficult to prove their direct involvement in the leaf morphology definition. In contrast, the multifunctional role of the Elongator complex represents a well-studied topic. This complex is constituted of six subunits, each in two copies. ELP1 and ELP2 are required for correct assembly, ELP3 is the catalytic subunit, and ELP4, ELP5, and ELP6 form an accessory complex [58,59]. Multiple cellular processes have proven the participation of this complex, including resistance to oxidative stress, cell cycle progression, and root and leaf development [60]. Notably, several authors were able to demonstrate that the *AtELP* mutants for the single or multiple subunits developed serrated and curly leaves [60,61,62]. Although it remains unclear whether a direct association between the nonsynonymous mutations found in ELP1/ELO2 and the curly leaves characterizing *C. endivia* var. *crispum* exists*,* this gene should be taken into consideration in future studies for a better understanding of the underlying mechanisms behind this phenotypic trait.

A new scenario that unravels the connection between the curly trait and the newly identified genomic SNP-rich region discriminating the two main cultivar biotypes of *C. endivia* is now possible.

## 5. Conclusions

In conclusion, the results have demonstrated the advantages of using a molecular, genome-wide approach to distinguish phenotypically similar breeding stocks. We first documented the inefficiency of the endive genotyping based on heterologous SSR markers (derived from *C. intybus*) due to both the reduced interspecific transferability and the low polymorphism rate. Then, we were able to discriminate the 32 endive lines using 4621 SNP markers and to predict the two main cultivar types (*C. endivia* var. *latifolium* and var. *crispum*) based on a subset of 50 single-nucleotide variants. In the context of distinctiveness, uniformity and stability DUS testing, our findings may have great implications for the official procedures for new variety registration and for the resolution of legal cases due to the fraudulent use or accidental exchange of plant materials. Finally, taking advantage of the newly produced SNP panels, we were able to identify and narrow down a genomic window that is particularly rich in discriminating markers; the gene(s) responsible for the leaf morphology may be located within this window.

## Figures and Tables

**Table 1:** Sequence of the primer pairs of 14 simple sequence repeats (SSR) marker loci selected from leaf chicory (*C. intybus*) that produced amplicons that were also in endive (*C. endivia*). The data include the original ID, SSR linkage group (LG), motif, tailed primers used (PAN1, PAN2, PAN3 or M13), and multiplex to which the SSR marker locus belongs. All the microsatellites used in this study were derived from Patella et al. [15].

ID	LG	Motif		Primer Sequence and Tail	Multiplex
M2.4	2	(GA)_25_	F	[PAN3]CCAACGGATACCAAGGTGTT	1
			R	AACCGCACGGGTTCTATG	
M2.5	2	(CT)_5_CC(CT)_13_ TT(CT)_5_	F	[PAN1]GTGCCGGTCTTCAGGTTACA	1
			R	CGCCTACCGATTACGATTGA	
M3.7	3	(CT)_22_	F	TTCGAGTCTTGCCTTAATTGTT	1
			R	[PAN1]CAGACGACCTTACGGCAACT	
M4.10a	4	(CT)_22_	F	[PAN2]CATCACCTTCACGAAAAGCA	1
			R	CGAAGACCATCCATCACCA	
M4.11a	4	(CT)_12_N_5_(CA)_11_	F	[PAN3]GAAGGAACCTATGAACCAACCACTCA	1
			R	GTTTTGAGCCTGAGCCAGA	
M1.3	1	(CT)_17_	F	[PAN3]TGGAGAAAAATGAAGCAC	2
			R	GAATGAGTGAGAGAATGATAGGG	
M5.13	5	(CT)_23_	F	[M13]AGGCATAAAGAGGTGTGG	2
			R	TCAAACATGAAAACCGCTC	
M6.17	6	(CA)_8_(CT)_18_	F	CGTGTCCAAACGCAAACATTAT	2
			R	[PAN2]GCACAATTTTCCTACCACTTATCC	
M5.14	5	(TC)_11_	F	[M13]AAAGTCACACATCGCATTTCCT	2
			R	GTAGCAGCAGCAGCCATCTT	
M4.11b	4	(TG)_5_CG(TG)_7_	F	[M13]GCCATTCCTTTCAAGAGCAG	2
			R	AACCCAAAACCGCAACAATA	
M3.9	3	(CA)_12_	F	CTGCTATGGACAGTTCCAGT	3
			R	[PAN3]CAATTCAGTTGTGATAGACGC	
M7.20	7	(CT)_31_	F	[PAN2]ACACTCACTCACACTCCGTAA	3
			R	GTCATGATGGCGTAAAAGTC	
M6.18	6	(CT)_16_	F	[PAN3]CTCAACGAATGCTTTGGACA	3
			R	CCTCGCGGTAGCTTATTGTT	
M2.6	2	(CT)_26_	F	GGAGCAGGTAGAGTCCCATC	3
			R	[PAN1]CGTTTGAAAATTTATACCAAAATG	

## Supplementary Materials

The following are available online at https://www.mdpi.com/2073-4425/11/4/462/s1, Figure S1: Definition of the number of ancestral parental lines based on the SSR marker dataset. The mean ∆K is calculated as |L” (K)|/(SD(L(K)), following Evanno et al. [22]. The blue line represents the ∆K values, Figure S2: Pairwise genetic similarity matrix of 32 endive lines (reported as percentages) based on Jaccard’s coefficient. The high genetic similarity values are labeled in red, the low values in green, and intermediate values are colored on a scale from red to green. Moreover, the percentage of observed homozygosity (Ho) of the 32 plant accessions is reported, Figure S3: Definition of the number of ancestral parental lines based on the SNP markers. The mean ∆K is calculated as |L” (K)|/(SD(L(K)), following Evanno et al. [22]. The blue line represents the ∆K values, Table S1: Microsatellite primer tails and dyes. List of the primer tails used with their sequences and corresponding dyes. Table S2: Validation of SNP primer pairs. List of the primer pairs of 6 SNP markers with their sequences and corresponding sizes. Table S3: PIC values for each locus found across 32 elite materials, Table S4: List of 555 RAD-seq reads mapped over the codifying loci of the 9 linkage groups (LGs) of *Lactuca sativa* and their hypothetical functions based on matches with the Arabidopsis protein database (TAIR database).
