# Supplementary material for: Genotyping by RAD Sequencing Analysis Assessed the Genetic Distinctiveness of Experimental Lines and Narrowed down the Genomic Region Responsible for Leaf Shape in Endive (Cichorium endivia L.)
Source: Genes (Basel). 2020 Apr 23;11(4):462. doi: 10.3390/genes11040462 (PMC7231076; doi:10.3390/genes11040462)
Supplement: Supplementary file 1 [file genes-11-00462-s001.zip › Supplementary Figures.docx]

Genotyping by RAD sequencing analysis assessed the genetic distinctiveness of experimental lines and narrowed down the genomic region responsible for leaf shape in endive (*Cichorium endivia* L.)

Alice Patella ^1§^, Fabio Palumbo ^1§^, Samathmika Ravi ^1^, Piergiorgio Stevanato ^1^ and Gianni Barcaccia ^1*^

^1^ Department of Agronomy, Food, Natural resources, Animals and Environment (DAFNAE), University of Padova, 35020 Legnaro PD, Italy; alice.patella@phd.unipd.it (A.P.); fabio.palumbo@unipd.it (F.P.); samathmikaravi@gmail.com (S.R.), stevanato@unipd.it (P.S.);

**§** These authors equally contributed to this work and share the co-first authorship

***** Correspondence: gianni.barcaccia@unipd.it (G.B.)

**Supplementary Figures**


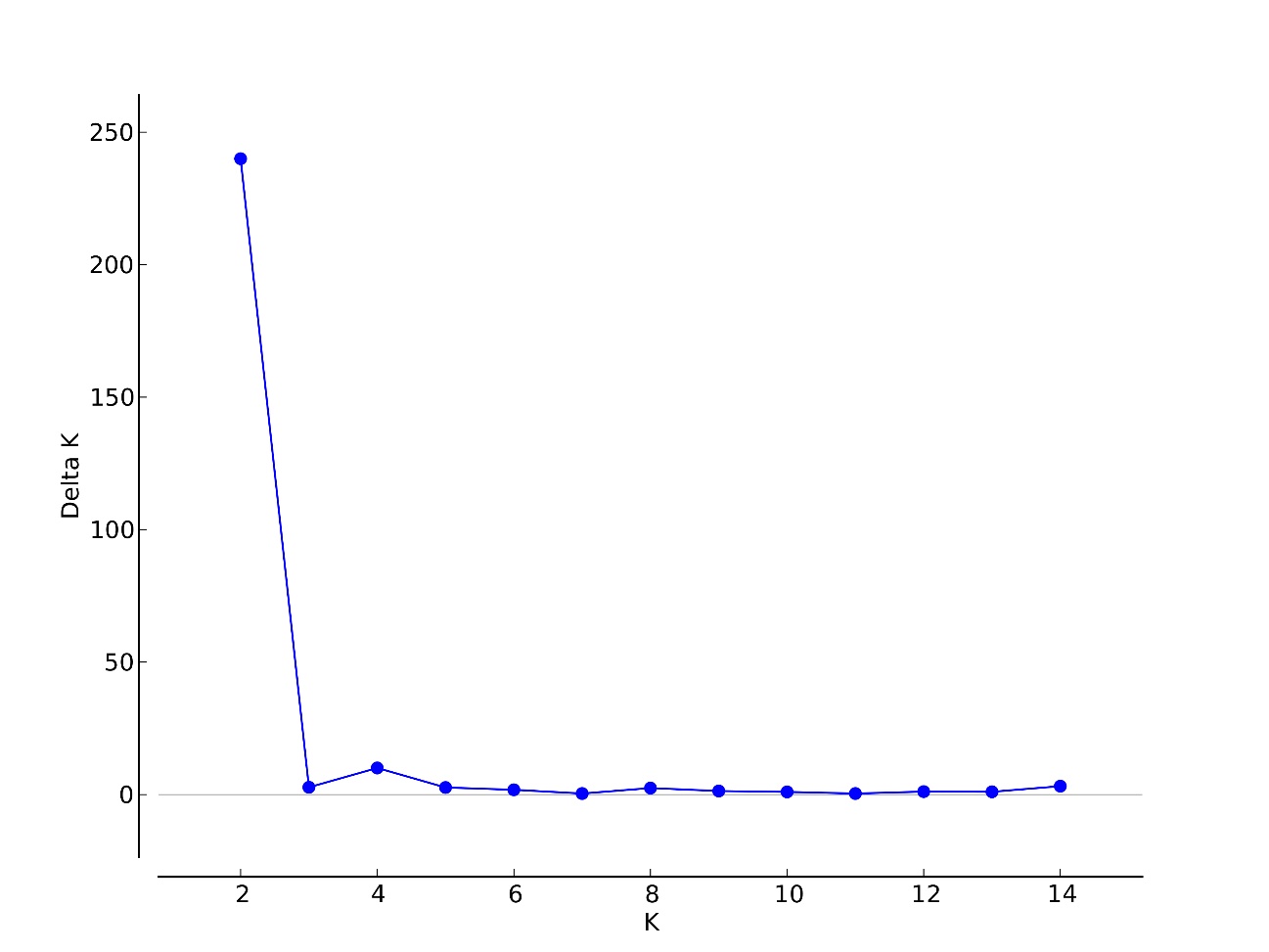


**Figure S1.** Definition of the number of ancestral parental lines based on the SSR marker dataset. The mean ∆K is calculated as |L” (K)|/(SD(L(K)), following Evanno *et al.* [22]. The blue line represents the ∆K values.

**
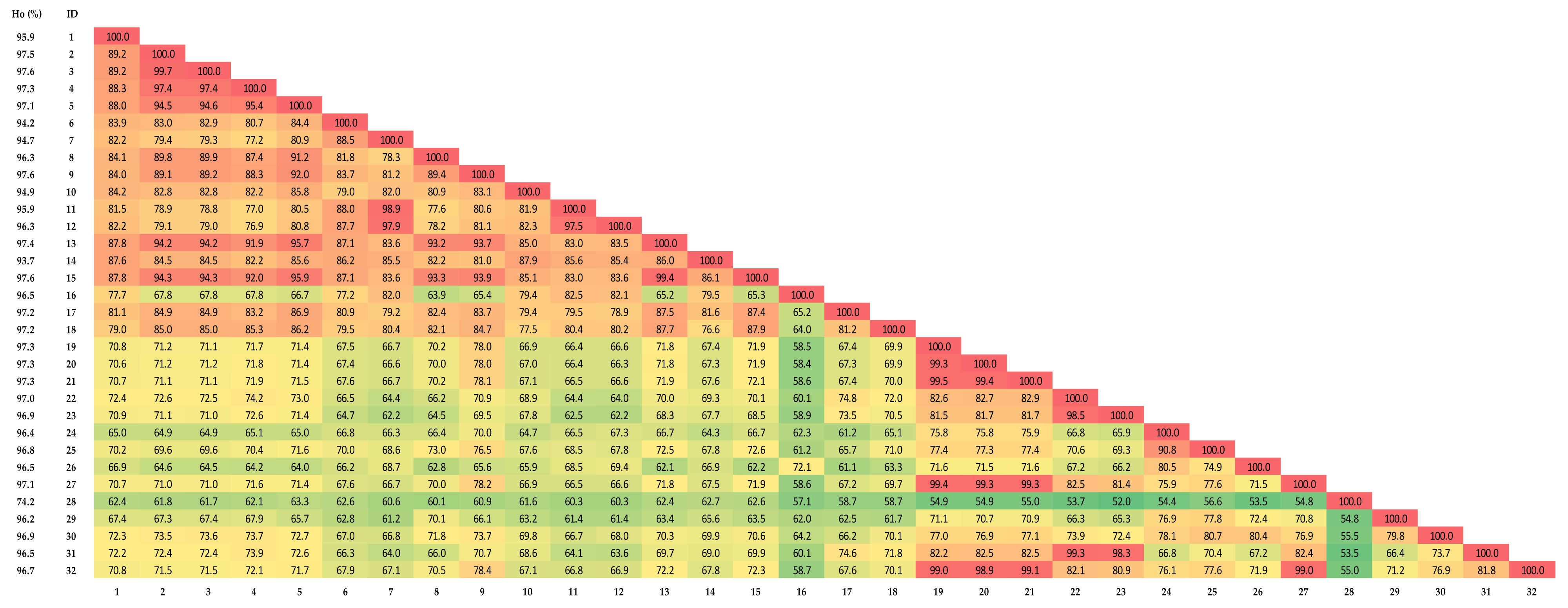
**

**Figure S2.** Pairwise genetic similarity matrix of 32 endive lines (reported as percentages) based on Jaccard’s coefficient. The high genetic similarity values are labeled in red, the low values in green, and intermediate values are colored on a scale from red to green. Moreover, the percentage of observed homozygosity (Ho) of the 32 plant accessions is reported.


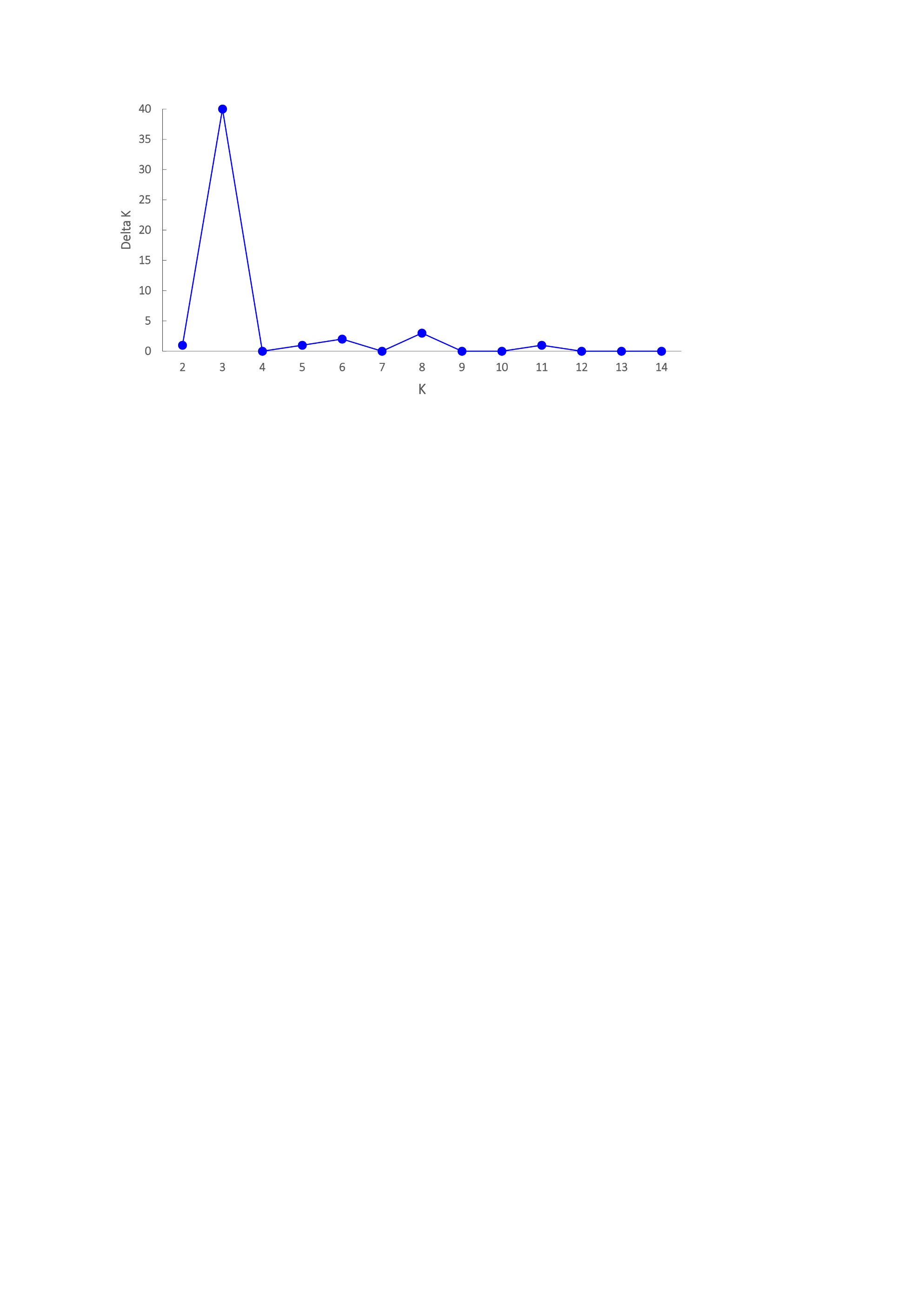
**Figure S3.** Definition of the number of ancestral parental lines based on the SNP markers. The mean ∆K is calculated as |L” (K)|/(SD(L(K)), following Evanno *et al.* [22]. The blue line represents the ∆K values.
