# Supplementary material for: Genotyping by RAD Sequencing Analysis Assessed the Genetic Distinctiveness of Experimental Lines and Narrowed down the Genomic Region Responsible for Leaf Shape in Endive (Cichorium endivia L.)
Source: Genes (Basel). 2020 Apr 23;11(4):462. doi: 10.3390/genes11040462 (PMC7231076; doi:10.3390/genes11040462)
Supplement: Supplementary file 1 [file genes-11-00462-s001.zip › Supplementary Tables.docx]

Genotyping by RAD sequencing analysis assessed the genetic distinctiveness of experimental lines and narrowed down the genomic region responsible for leaf shape in endive (*Cichorium endivia* L.)

Alice Patella ^1§^, Fabio Palumbo ^1§^, Samathmika Ravi ^1^, Piergiorgio Stevanato ^1^ and Gianni Barcaccia ^1*^

^1^ Department of Agronomy, Food, Natural resources, Animals and Environment (DAFNAE), University of Padova, 35020 Legnaro PD, Italy; alice.patella@phd.unipd.it (A.P.); fabio.palumbo@unipd.it (F.P.); samathmikaravi@gmail.com (S.R.), stevanato@unipd.it (P.S.);

**§** These authors equally contributed to this work and share the co-first authorship

***** Correspondence: gianni.barcaccia@unipd.it (G.B.)

**Supplementary Tables**

**Table S1.** Microsatellite primer tails and dyes. List of the primer tails used with their sequences and corresponding dyes.

| **Universal primer** | **Sequence 5'-3'** | **Dye** |
| --- | --- | --- |
| M13 | TTGTAAAACGACGGCCAGT | 6-FAM |
| PAN1 | GAGGTAGTTATTGTGGAGGAC | VIC |
| PAN2 | GGAATTAACCGCTCACTAAAG | NED |
| PAN3 | TGTAGAAAGACGAAGGGAAGG | PET |

**Table S2.** Validation of SNP primer pairs. List of the primer pairs of 6 SNP markers with their sequences and corresponding sizes.

| **Reference reads** |  | **Primer sequence** | **Sequence  length (bp)** |
| --- | --- | --- | --- |
| 3614 | F | CCAGCTAACAAAAGCCATTGCA | 707 |
|  | R | TCCATTTGATGTTGAAGTTCCTC |  |
| 3508 | F | CGCTAAAATCGCCACTGTTTG | 543 |
|  | R | ACACGAAAGACTTCCCCTTC |  |
| 4087 | F | TATGAATAGTCGGCTGGGGA | 396 |
|  | R | CAATTACTGCAATTGATGGTAC |  |
| 857 | F | CTCAGGGTCAAGATTCGGGT | 316 |
|  | R | ACAGTGTAGCTCTTGATGTTGT |  |
| 414 | F | TTGTGGTGTGGGCTTAGTGA | 182 |
|  | R | GGTGATGGGAATCGTGGC |  |
| 1310 | F | TCATTGTGGTCGGGAATCTTG | 438 |
|  | R | CTGAGCAACAAGCATTTGACT |  |

**Table S3.** PIC values for each locus found across 32 elite materials.

| **ID** | **PIC** |
| --- | --- |
| M2.4 | 0.61 |
| M2.5 | 0.7 |
| M3.7 | 0.49 |
| M4.10a | 0 |
| M4.11a | 0.65 |
| M1.3 | 0 |
| M5.13 | 0.47 |
| M6.17 | 0.44 |
| M5.14 | 0 |
| M4.11b | 0.58 |
| M3.9 | 0 |
| M7.20 | 0 |
| M6.18 | 0 |
| M2.6 | 0.42 |

**Table S4.** List of 555 RAD-seq reads mapped over the codifying loci of the 9 linkage groups (LGs) of *Lactuca sativa* and their hypothetical functions based on matches with the Arabidopsis protein database (TAIR database).

**(See Excel File)**

**Table S5.** SNPs detected by an additional Sanger-based validation step performed on the six reads carrying nonsynonymous mutations and discriminating the two main cultivar types. For each original RAD-seq read, the putative genes to which it belongs, the position and type of nucleotide variants identified through Sanger sequencing (escarole/curly endive) and the amplicon length (bp) are reported. Nonsynonymous SNPs are reported in red; nucleotide variants found in exon regions are shown in bold, and stars indicate SNPs already identified through RAD sequencing.

| **Reference  reads** | **Putative gene** |  | **Nucleotide variants identified through Sanger sequencing** | | | | | | | | | | | | | | **Amplicon length (bp)** | | |
| --- | --- | --- | --- | --- | --- | --- | --- | --- | --- | --- | --- | --- | --- | --- | --- | --- | --- | --- | --- |
| 3614 | CASP Like 5A2 | Pos | 218 | 249 | 254 | 274 | **367** | 478 | 493 | 512 | 521 | 528 | 557 | 565 | 571 | **654*** | | 707 |  |
|  |  | Var | C > T | C > T | A > C | A > G | **C > T** | TA > AG | G > A | C > T | A > T | -- > TT | - > ATTGATG | G > A | T > C | **T > A** | |  |  |
| 3508 | elongator complex 1  (ELP1,) | Pos | **114** | **238** | **252** | **261** | **361*** | **368** | **435** | **465** | **471** |  |  |  |  |  | | 543 |  |
|  |  | Var | **A > C** | **A > G** | **A > G** | **T > A** | **A > G** | **A > G** | **T > C** | **CT > TC** | **A > T** |  |  |  |  |  | |  |  |
| 4087 | Phosphatidylglycerol phosphate synthase 1 | Pos | **13** | 84* | 112 | 123 | 229 | **353** |  |  |  |  |  |  |  |  | | 396 |  |
|  |  | Var | **G > C** | T > A | T > A | - > G | A > T | **C > G** |  |  |  |  |  |  |  |  | |  |  |
| 857 | 26S proteasome regulatory subunit N10 | Pos | **44** | **80** | 168 | **194*** |  |  |  |  |  |  |  |  |  |  | | 316 |  |
|  |  | Var | **T > C** | **T > C** | T > G | **T > C** |  |  |  |  |  |  |  |  |  |  | |  |  |
| 414 | Aquaporin PIP1-1-Related | Pos | - |  |  |  |  |  |  |  |  |  |  |  |  |  | | 182 |  |
|  |  | Var | - |  |  |  |  |  |  |  |  |  |  |  |  |  | |  |  |
| 1310 | cysteine protease ATG4 | Pos | - |  |  |  |  |  |  |  |  |  |  |  |  |  | | 438 |  |
|  |  | Var | - |  |  |  |  |  |  |  |  |  |  |  |  |  | |  |  |
